# Effect of housing arrangement on fecal-oral transmission of avian hepatitis E virus in chicken flocks

**DOI:** 10.1186/s12917-017-1203-4

**Published:** 2017-09-07

**Authors:** Baoyuan Liu, Yani Sun, Yiyang Chen, Taofeng Du, Yuchen Nan, Xinjie Wang, Huixia Li, Baicheng Huang, Gaiping Zhang, En-Min Zhou, Qin Zhao

**Affiliations:** 1 Department of Preventive Veterinary Medicine, College of Veterinary Medicine, Northwest A&F University, 22 Xinong Road, Yangling, Shaanxi China; 2 Scientific Observing and Experimental Station of Veterinary Pharmacology and Veterinary Biotechnology, Ministry of Agriculture, Yangling, Shaanxi China; 3 Department of Preventive Veterinary Medicine, College of Veterinary Medicine, Henan Agriculture University, Zhengzhou, Henan 450002 China

**Keywords:** Avian HEV, Housing arrangement, Virus transmission, Prevention

## Abstract

**Background:**

Avian hepatitis E virus (HEV) infection is common in chicken flocks in China, as currently no measures exist to prevent the spread of the disease. In this study, we analyzed the effect of caged versus cage-free housing arrangements on avian HEV transmission. First, 127 serum and 110 clinical fecal samples were collected from 4 chicken flocks including the two arrangements in Shaanxi Province, China and tested for HEV antibodies and/or virus. Concurrently, 36 specific-pathogen-free chickens were divided equally into four experimental living arrangement groups, designated cage-free (Inoculated), caged (Inoculated), cage-free (Negative) and caged (Negative) groups. In caged groups, three cages contained 3 chickens each. Three chickens each from cage-free (Inoculated) and caged (Inoculated) groups (one chicken of each cage) were inoculated by cutaneous ulnar vein with the same dose of avian HEV, respectively. The cage-free (Negative) and caged (Negative) groups served as negative control. Serum and fecal samples were collected at 1 to 7 weeks post-inoculation (wpi) and liver lesions were scored at 7 wpi.

**Results:**

The results of serology showed that the avian HEV infection rate (54.10%) of the cage-free chickens was significantly higher than the one (12.12%) for caged chickens (*P* < 0.05). Also, the rate of detection of avian HEV RNA in the clinical fecal samples was significantly higher in the cage-free (22.80%, 13/57) than caged birds (5.66%, 3/53). Moreover, under experimental conditions, the infected number of uninoculated cage-free chickens (6) was significantly higher than the one for the uninoculated caged birds (2), as evidenced by seroconversion, fecal virus shedding, viremia and gross and microscopic liver lesions.

**Conclusions:**

These results suggest that reduction of contact with feces as seen in the caged arrangement of housing chickens can reduce avian HEV transmission. This study provides insights for prevention and control of avian HEV infection in chicken flocks.

## Background

Avian hepatitis E virus (HEV) is the causative agent of big liver and spleen disease and hepatitis-splenomegaly syndrome in chickens [1, 2]. The disease is characterized by increased mortality (1%–4%), a decrease in egg production (10%–40%) and enlarged livers and spleens in broiler breeder and laying hens aged 30–72 weeks [3, 4]. The virus can also cause mild clinical disease in chicken flocks, characterized by a decrease in egg production when the external environment is altered (such as with changes in climate and feed) and/or if infection occurs concurrently with other pathogen infections [5]. Although vertical transmission of the virus has been reported recently [6], avian HEV is believed to be transmitted mainly by the fecal-oral route in flocks [1, 7, 8]. To date the virus has been reported to be present in many countries [1, 2, 8–13].

Avian HEV is a non-enveloped single-stranded, positive sense RNA virus that is classified as an *Orthohepevirus* B species within the *Hepeviridae* family [14]. Sharing only 48% identity with human and swine HEVs, the avian HEV genome is approximately 6.6 kb in size and consists of three open-reading frames (ORFs) and two noncoding regions [15]. The ORF2 gene encodes a capsid protein containing the major viral epitopes; this capsid protein thus serves as the target for serological diagnosis and vaccine design [8, 16–18]. Although avian HEV strains have been divided into 4 major genotypes [19], they all belong to a single serotype [20].

In China in 2010, an avian HEV strain infecting a broiler breeder chicken flock exhibiting hepatitis-splenomegaly syndrome was isolated and characterized [11]. Subsequently, several serological surveys have indicated that avian HEV infection is widespread in chicken flocks in China [16, 21]. However, due to the lack of effective vaccines and drugs, no practical measures yet exist to prevent and control the disease, which hampers healthy development of poultry. Ultimately, blocking fecal-oral transmission should prevent the spread of virus infection [22], especially since this route has been shown to be the main avian HEV transmission route in chicken flocks [1, 7, 8]. Therefore, we evaluated the efficacy of disease control through inhibition of chicken contact with feces.

## Results

### Detection of avian HEV antibodies and RNA in clinical samples

**Fig. 1:**
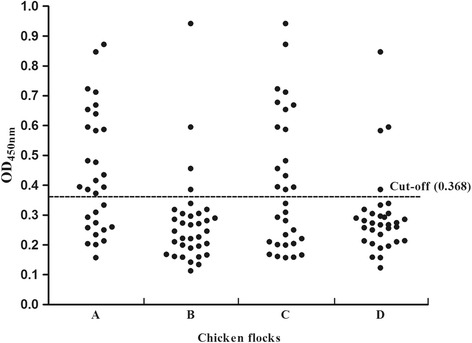
Distribution of OD_450nm_ values of sera from the caged and cage-free arrangements using indirect ELISA. The dotted line represents the cut-off value (0.368)

The overall anti-avian HEV seropositivity rate was 32.28% (41/127), while the seropositivity rates for flocks A, B, C and D were 60% (18/30), 11.76% (4/34), 48.39% (15/31) and 12.5% (4/32), respectively (Table 1). The OD_450nm_ value distributions of the serum samples tested for antibody detection using indirect ELISA are shown in Fig. 1. For the two types of living arrangements, the positive rates of cage-free and caged chickens were 54.10% (33/61) and 12.12% (8/66), respectively. Statistical analyses showed that the difference in positive rates based on the type of living arrangements was significant (*P* < 0.05).

**Table 1:** Detection of avian HEV antibodies and RNA in sera and feces from 4 layer flocks

Chicken flock	Scale	Breed	Housing arrangements	Serum samples			Fecal samples		
				Detected number	Sum	Positive rate	Detected number	Sum	Positive rate
A	2000	Hy-Line Brown	Cage-free	18	30	60%	7	28	25%
B	10,000	Hy-Line Brown	Caged	4	34	11.76%	1	25	4%
C	3000	Hy-Line Brown	Cage-free	15	31	48.39%	6	29	20.69%
D	8000	Hy-Line Brown	Caged	4	32	12.5%	2	28	7.14%

**Fig. 2:**
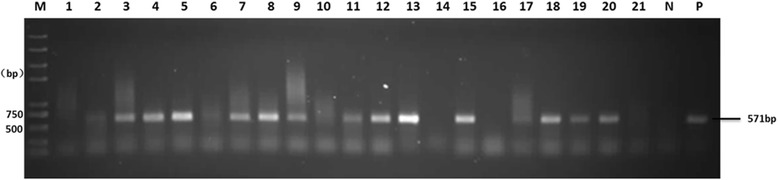
Agarose gel analysis of PCR products for the avian HEV partial ORF1 gene from faeces. M:Trans2K® Plus II DNA marker;1–8:A flock;9–10:B flock;11–18:C flock;19–21:D flock;N:Ultrapure water;P:A bile sample containing avian HEV

Consequently, agarose gel analysis showed that 16 of the samples exhibited bands of the expected size of 571 bp (Fig. 2). The 16 positive PCR products were sequenced and the sequences were analyzed using BLAST searches, nucleotide sequence alignments as well as phylogenetic analyses with known avian HEV sequences in the GenBank. The results showed that the 16 sequences shared high identity (82%–99%) with other known avian HEV strains from GenBank, indicating the 16 fecal samples contained avian HEV RNA. The total HEV positive rate of these fecal samples was 14.55% (16/110) and the positive rates of A, B, C and D flocks were 25% (7/28), 4% (1/25), 20.69% (6/29) and 7.14% (2/28), respectively (Table 1). For the two living arrangements, the positive infection rates of the cage-free and caged groups were 22.80% (13/57) and 5.66% (3/53), respectively. Statistical analyses also showed that the difference in positive rates between cage-free and caged chickens for avian HEV RNA in the fecal samples was significant (*P* < 0.05).

### Avian HEV antibody seroconversion in experimental chickens

**Fig. 3:**
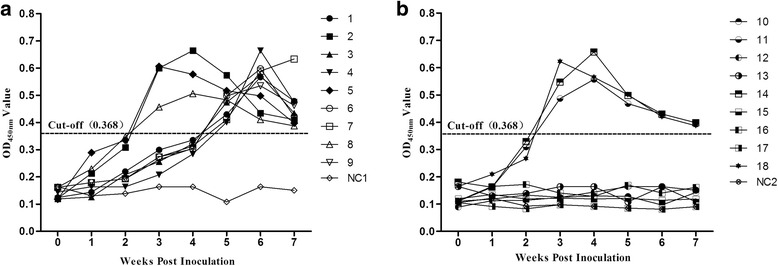
Seroconversion to avian HEV in the experimental chickens from the cage-free and caged arrangements. **a**: Cage-free (Inoculated) groups;(**b**): Caged (Inoculated) groups. The dotted line represents the cut-off values of the indirect ELISA used for detection of the avian HEV antibodies in the serum samples. NC1: Cage-free (Negative) group;NC2: Caged (Negative) group. The week post-inoculation on 0 the X axis represents the period prior to inoculation

Prior to inoculation, all SPF chickens were shown to be seronegative for avian HEV (Fig. 3). Based on the cut-off value (0.368) of the indirect ELISA, anti-avian HEV IgG antibodies were detected in the 6 inoculated chickens of both cage-free (Inoculated) and caged (Inoculated) groups (Nos. 2, 5, 8, 11, 14 and 18) at 3 wpi (Fig. 3). At the end of the experiment (7 wpi), all 6 chickens were still positive for anti-avian HEV antibodies (Fig. 3). The remaining 6 uninoculated chickens in cage-free (Inoculated) group seroconverted to IgG anti-avian HEV antibodies by 5 wpi and were still positive at 7 wpi (Table 2, Fig. 3). However, in caged (Inoculated) group, all remaining chickens from the three cages remained seronegative throughout the study (Table 2, Fig. 3). In the cage-free (Negative) and caged (Negative) groups, all the chickens were seronegative throughout the study (Fig. 3).

**Table 2:** Number of the uninoculated chickens infected by avian HEV in the cage-free and caged groups

Housing arrangements	Seroconversion to avian HEV	Fecal virus shedding	Viremia	Gross lesions	Microscopic lesions
Cage-free (Inoculated)	6/6	6/6	6/6	5/6	5/6
Caged (Inoculated)	0/6	2/6	1/6	0/6	1/6
Cage-free (Negative)	0/6	0/6	0/6	0/6	0/6
Caged (Negative)	0/6	0/6	0/6	0/6	0/6

The chickens were infected as evidenced by seroconversion, fecal virus shedding, viremia and gross and microscopic lesions of livers. The number was shown as positive number/total number

### Detection of avian HEV RNA in fecal and serum samples from experimental chickens

The fecal and serum samples from all chickens were negative for avian HEV RNA at pre-inoculation (Table 3). Fecal virus shedding was detected in the 5 of 6 inoculated chickens from cage-free (Inoculated) and caged (Inoculated) groups at 1 wpi (Table 3) while all 6 inoculated chickens were positive at 2 wpi (Table 3). At 5 wpi, there was no more shedding of virus in the feces of these chickens (Table 3). For the uninoculated chickens, fecal virus shedding was first detected in 2 of 6 chickens in cage-free (Inoculated) group at 3 wpi; at 6 wpi all 6 chickens were positive (Tables 2 and 3). However, in caged (Inoculated) group, only two uninoculated chickens housed in the two cages containing the inoculated chickens exhibited fecal virus shedding at 6 wpi (Tables 2 and 3). The remaining four uninoculated chickens in the cages were all negative (Table 3). Viremia was also first observed in inoculated chickens at 1 wpi (Table 3). All 6 inoculated chickens of cage-free (Inoculated) and caged (Inoculated) groups exhibited viremia at 3 wpi (Table 3). For the uninoculated chickens in cage-free (Inoculated) group, viremia was observed in 2 of 6 chickens at 3 wpi, 4/6 chickens at 4 wpi, 3/6 chickens at 5 wpi and 3/6 chickens at 6 wpi (Tables 2 and 3). However, in caged (Inoculated) group, viremia was only observed in 1/6 uninoculated chickens at 6 wpi (Tables 2 and 3). In cage-free (Negative) and caged (Negative) groups, all chickens remained negative throughout the experiment (Data not shown).

**Table 3:** Fecal virus shedding and viremia of all the chickens in the cage-free (Inoculated) and caged (Inoculated) groups

Housing arrangements	Chicken No.	Detection of fecal virus shedding /viremia from different weeks post inoculation							
		0	1	2	3	4	5	6	7
Cage-free (Inoculated)	1	−/−	−/−	−/−	+/+	−/+	+/−	+/	−/−
	**2**	**−/−**	**+/+**	**+/−**	**−/+**	**+/−**	**−/−**	**−/−**	**−/−**
	3	−/−	−/−	−/−	−/−	−/−	+/+	+/+	−/−
	4	−/−	−/−	−/−	+/+	+/+	−/−	+/+	−/−
	**5**	**−/−**	**+/−**	**+/+**	**−/+**	**+/−**	**−/−**	**−/−**	**−/−**
	6	−/−	−/−	−/−	−/−	−/−	−/+	+/−	−/−
	7	−/−	−/−	−/−	−/−	−/+	+/+	+/−	−/−
	**8**	**−/−**	**+/+**	**+/−**	**+/+**	**+/−**	**−/−**	**−/−**	**−/−**
	9	−/−	−/−	−/−	−/−	+/+	+/−	+/+	−/−
Caged (Inoculated)	10	−/−	−/−	−/−	−/−	−/−	−/−	−/−	−/−
	**11**	**−/−**	**+/+**	**+/+**	**−/+**	**+/−**	**−/−**	**−/−**	**−/−**
	12	−/−	−/−	−/−	−/−	−/−	−/−	−/−	−/−
	13	−/−	−/−	−/−	−/−	−/−	−/−	+/−	−/−
	**14**	**−/−**	**+/−**	**+/+**	**−/+**	**+/−**	**−/−**	**−/−**	**−/−**
	15	−/−	−/−	−/−	−/−	−/−	−/−	−/−	−/−
	16	−/−	−/−	−/−	−/−	−/−	−/−	+/+	−/−
	17	−/−	−/−	−/−	−/−	−/−	−/−	−/−	−/−
	**18**	**−/−**	**−/+**	**+/−**	**+/+**	**+/−**	**−/−**	**−/−**	**−/−**

The bold numbers represent the inoculated chickens. For the caged group, the Nos 10–12 were in the same cage, the Nos 13–15 in the same cage and the Nos 16–18 in the same cage. “+” represents the chickens’ positive for fecal virus shedding and viremia

The positive PCR products from serum samples and fecal swabs of inoculated and uninoculated chickens of cage-free (Inoculated) and caged (Inoculated) groups were sequenced and BLAST results showed that the viruses recovered from the infected chickens originated from the original virus inoculum.

### Gross lesions

**Fig. 4:**
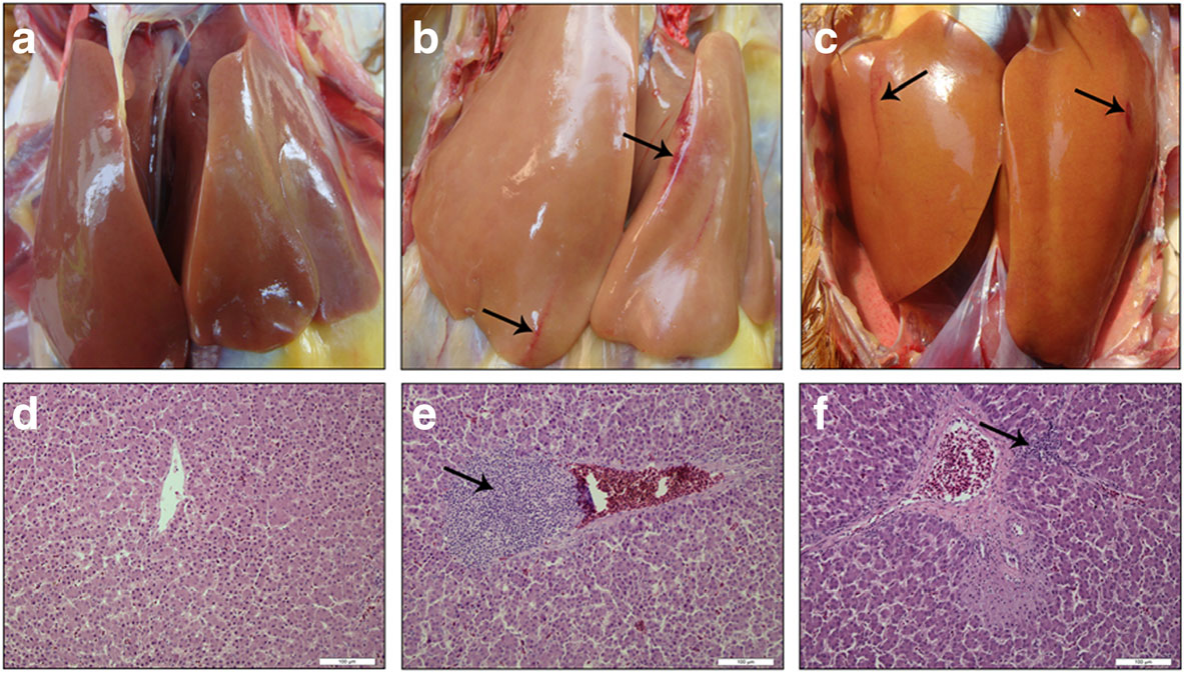
Gross lesions and microscopic lesions of the livers from experimental chickens. Subcapsular hemorrhages (**a**, **b** and **c**) and lymphocytic periphlebitis (**d**, **e** and **f**) are indicated by arrows. **a** and **d**: uninfected chickens in cage-free (Negative) group; (**b**) and (**e**): inoculated chickens from cage-free (Inoculated) group; (**c** and **f**): chickens infected by contact with feces from cage-free (Inoculated) group. Tissues were stained with hematoxylin and eosin

Gross lesions were observed clearly in the 6 inoculated chickens from cage-free (Inoculated) and caged (Inoculated) groups at 7 wpi. Subcapsular hemorrhages were observed in the livers of the 6 chickens and several hemorrhagic spots and regions were also observed in livers (Fig. 4). In cage-free (Inoculated) group, hemorrhagic spots and regions were observed in 5 of the 6 remaining chickens (Table 2). However, in caged (Inoculated) group, gross lesions in the 6 remaining chickens were not evident (Table 2). In addition, compared with the uninfected chickens, the liver/body weight ratios from the 6 inoculated chickens and 2 contact-infected chickens of cage-free (Inoculated) were significantly higher (Table 4). In cage-free (Negative) and caged (Negative) groups, all chickens had no evident liver lesions.

**Table 4:** Scoring of microscopic liver lesions and liver/body weight ratios in all the chickens. For caged group, the Nos 10–12, 13–15, 16–18, 28–30, 31–33 and 34–36 chickens were kept separately in the same cage

Housing arrangements	No. of chickens (score ^a^, liver/body weight ratio ^b^)								
Cage-free (Inoculated)	1(1, 23.16)	**2(4, 40.92)**	3(2, 23.44)	4(2, 32.19)	**5(4, 37.98)**	6(3, 33.25)	7(2, 24.01)	**8(4, 36.78)**	9(2, 23.48)
Caged (Inoculated)	10(0, 24.84)	**11(4, 36.47)**	12(1, 23.39)	13(0, 22.08)	**14(4, 35.88)**	15(0, 24.12)	16(2, 23.98)	17 (1, 22.68)	**18(4, 38.11)**
Cage-free (Negative)	19(0, 23.98)	20(0, 24.12)	21(1, 22.65)	22(0, 23.65)	23(1, 24.09)	24(0, 23.51)	25(0, 22.98)	26(1, 23.65)	27(0, 24.01)
Caged (Negative)	28(1, 22.99)	29(0, 25.01)	30(0, 23.98)	31(0, 24.19)	32(1, 24.67)	33(2, 24.88)	34(0, 22.87)	35(1, 23.13)	36(0, 24..35)

^a^ Liver lesion scores ranged from 0 to 4 (0, no lesions; 1, <5 foci; 2, 5 to 8 foci; 3,9 to 15 foci; 4,>15 foci)

^b^ Liver to body weight ratio was calculated by (liver weight)/(body weight) × 100

The liver of each chicken was weighed and the liver/body weight ratios were calculated. Compared with the uninfected chickens, the mean ratios from the 6 inoculated chickens (bold) and 2 contact-infected chickens (Nos.4 and 6) were significantly higher(*P* < 0.05)

### Microscopic lesions

Microscopically, hepatic lesions were all observed in the livers of the 6 inoculated chickens from cage-free (Inoculated) and caged (Inoculated) groups, showing severe lymphocytic portal phlebitis and periphlebitis (Fig. 4). For the uninoculated chickens in cage-free (Inoculated) group, five contact-infected chickens exhibited mild lymphocytic phlebitis in their livers (Fig. 4). However, in caged (Inoculated) group, microscopic lesions were only evident in 1 of 6 uninoculated chickens. The data on microscopic lesions in livers from all the chickens are summarized (Table 4) and show that the lesion scores of the infected chickens kept in cage-free conditions were significantly higher than that of the caged chickens (Table 4). In cage-free (Negative) and caged (Negative) groups, all chickens had no evident liver microscopic lesions.

## Discussion

Avian HEV causes not only big liver and spleen disease, but also subclinical infections in chickens [5]. The virus infection is endemic in many countries and has resulted in serious economic losses in the poultry industries in some countries [1, 2, 8–13]. Avian HEV was first characterized in 2010 in China and subsequent serological and molecular epidemiological investigations indicated that it is now common in Chinese chicken flocks [11, 16, 21]. In this study, we detected anti-avian HEV antibodies and avian HEV RNA in four seemingly healthy chicken flocks of Shaanxi Province, China. The positive rates for antibodies (32.28%) and viral RNA (14.55%) suggest that avian HEV infection is common in these 4 flocks from Shaanxi Province, which aligns with HEV detection rates in other provinces in China [16].

In China, two major chicken housing arrangements are used in the poultry industry. One is the cage-free arrangement in which the birds are housed and fed in a shared region and can mingle and move freely about. Another is the caged condition, in which the birds are housed and fed in different cages and cannot mingle freely. Previous studies have documented that resistance to diseases varies for animals kept under different living conditions [23, 24]. In this study the positive rates for avian HEV antibodies and RNA under both housing arrangements showed that the avian HEV infection rate was higher for cage-free compared to caged animals. Then, the animal experimental results also showed that the number of infected chickens present in the cage-free arrangement was greater than that for the caged group. The key point of difference between cage-free and caged chickens lies in whether or not chickens come into contact with feces of other chickens. Based on this difference and the known primary route of avian HEV transmission, we speculate that the reason for the higher rates in the cage-free arrangement was due to uninhibited contact between chickens and feces of their housing companions.

By 4 wpi, viremia was undetected in all 6 inoculated chickens of cage-free and caged groups while antibodies against avian HEV peaked at 3 to 4 wpi, coinciding with the disappearance of virus from the blood stream. After 1 week pi, fecal virus shedding also became undetectable. Some of the uninoculated chickens were observed to be infected and this could have occurred through contact with feces from the inoculated chickens. However, compared to the latter, they experienced a delay of 2 or more weeks before seroconversion, viremia and fecal virus shedding occurred. This observation can be explained by the fact that in the inoculated chickens which were infected intravenously, the virus would have invaded the bloodstream directly before getting to the liver where massive replication takes place. Subsequently, it would pass through the bile into the gastrointestinal tract before being excreted from the body [25]. However, for the uninoculated chickens infected by fecal-oral routes, a small quantity of virus replicates first in the intestinal tract before getting into the blood and liver. This procedure takes a long time before virus replication [25].

For the uninoculated animals in the caged group, two chickens in the two cages were infected. Since the 1 inoculated and 2 uninoculated chickens were housed in the same cage for 7 weeks, it is possible that some avian HEV-contaminated feces from the inoculated chicken may have adhered to the edges of the cage, and this could have been ingested by the uninoculated chickens through the fecal-oral route. In addition, compared with cage-free chickens infected after feces contact, the two HEV RNA positive caged chickens showed no avian anti-HEV antibody seroconversion and no evident gross liver lesions. Moreover, these results also suggest that the two infected chickens may have been infected during the late phase of the study or were infected with a very small amount of virus after contact with contaminated feces. To date, there are few reports about prevention and control measures for avian HEV infection in chicken flocks. Based on the results of this study, control of chicken excrement pollution could be reduced of minimize avian HEV infection. Moreover, the caged living arrangement exhibited greater prevention of HEV transmission than did the cage-free arrangement.

## Conclusions

Overall, we report that the rate of avian HEV infection in chicken flocks living in cage-free conditions was higher than that observed for caged birds. Next, using animal experiments, we confirmed that differences in rate of contact with feces can explain why the caged living arrangement was more effective than the cage-free arrangement for prevention of avian HEV infection. Therefore, this study provides some insights into the prevention and control of avian HEV infection in chicken flocks.

## Methods

### Clinical sample collection and processing

The profiles of four healthy chicken flocks in Shaanxi Province, China (A, B, C and D) that were used to provide 127 serum and 110 fecal samples for HEV testing are shown in Table 1.

Fecal samples were homogenized in 10% (*w*/*v*) sterile phosphate-buffered saline (PBS, pH 7.2). The fecal homogenates were clarified by centrifugation at 1000 *g* for 10 min at 4 °C and 200 μL supernatants were used for the detection of avian HEV RNA using reverse transcription-nested PCR (RT-nPCR). The serum samples were used for the detection of anti-avian HEV antibodies by indirect ELISA.

### Virus

An avian HEV infectious stock was produced by intravenously inoculating four 8-week-old specific-pathogen-free (SPF) chickens with 200 μL of a clinical bile sample containing avian HEV isolated from a chicken aged 35 weeks (CaHEV, GenBank accession no. GU954430). This avian HEV stock contained 10^4^ genomic equivalents (GE)/mL or 500 median chicken infective dose (CID_50_)/mL of the virus.

### Chickens

Thirty-six 8-week-old, SPF female chickens were purchased from Beijing Merial Vital Laboratory Animal Technology Company. All birds were negative for avian HEV antibodies and RNA.

### Animal experimental design

The 36 SPF chickens were randomly divided into 4 experimental groups, with 9 chickens per group. The chickens (Nos. 1 through 9) in cage-free (Inoculated) group were housed in a room with a floor space of 6 square meters and could regularly contact each other and their companions’ feces. The 9 chickens (Nos. 10 through 18) in the caged (Inoculated) group were divided into 3 cages and located in a room. Each cage had a footprint of 2 square meters per cage and housed 3 chickens. The 3 cages were placed closely spaced and side-by-side such that chickens in different cages could not contact each other freely, although chickens within each cage could contact one another. All 9 chickens in caged (Inoculated) group were not expected to contact feces because the feces dropped from the cages and were cleared quickly, although some feces may have adhered to the edges of the cage. The 9 chickens (Nos. 19 through 27) in cage-free (Negative) group were housed same as the cage-free (Inoculated) group and the chickens (Nos. 28 through 36) in the caged (Negative) group were arranged same as the caged (Inoculated) group. The temperatures in the four rooms ranged from 20 to 21 °C and 10/14 h (light/dark) cycle was given for chickens to get enough sleep. They had free access to water and a commercial starter diet without supplementation of antibiotics.

In cage-free (Inoculated) and caged (Inoculated) groups, six chickens (Nos. 2, 5, 8, 11, 14, and 18) were randomly selected for inoculation by cutaneous ulnar vein with 800 μL of avian HEV stock. In cage-free (Negative) and caged (Negative) groups, chickens served as uninoculated controls under cage-free and caged conditions, respectively. All chickens were monitored for avian HEV infection for 7 weeks and then were necropsied. The animal experiments were approved by the Animal Care and Use Committee of Northwest Agricultural & Forestry University (NWSUAF, Permit Number: AE189135) with adherence to NWSUAF guidelines during handling of all experimental animals.

### Animal experimental samples collection

Serum and fecal swab samples were collected prior to inoculation and weekly thereafter as described above. During each round of blood collection, the gloves, needles and gowns were changed to avoid introduction of cross-infection among the chickens by the procedures used. Serum samples were tested by indirect ELISA for anti-avian HEV IgG antibodies while serum and fecal samples were tested by RT-nPCR for avian HEV RNA. The indirect ELISA and RT-nPCR procedures for testing experimental samples were same methods used for clinical samples, as described below.

### Gross and microscopic hepatic lesions

During necropsies, gross pathological lesions in the liver of each chicken were evaluated and recorded. In addition, the liver of each chicken was weighed and the liver/body weight ratios were calculated. The liver tissues were also fixed in 10% neutral buffered formalin and processed for routine histological examination. Histopathological lesions in the liver were evaluated and scored according to a standard scoring system documented previously by Billam et al. [25]. Liver lesion scores ranged from 0 to 4 (0, no lesions; 1, <5 foci; 2, 5 to 8 foci; 3, 9 to 15 foci; 4, >15 foci).

### RT-nPCR to detect avian HEV RNA

The partial ORF1 gene of the avian HEV genome was amplified using RT-nPCR from both clinical and experimental fecal and serum samples [26]. Two nested sets of primers were used: external primer set DHF1, 5′-TGAGGGTTCGAGCTGACAG-3′ and DHR1, 5′-CATACGCCTCGTCCACAAT-3′; and internal primer set DHF2, 5′-CAGCAGCCATCCGCAAAC-3′ and DHR2, 5′-GGACGCCTGATGAACAACG-.3′, which were described previously by Dong et al. [26]. The sizes of the expected PCR products for the first and second round PCRs were 921 bp and 571 bp, respectively.

Total RNA was extracted from each 200 μL serum sample or 10% fecal suspension with TRIzol reagent (ThermoFisher Scientific, USA) according to the manufacturer’s instructions and was resuspended in 20 μL DNase-, RNase-, and proteinase-free water. Superscript® II Reverse Transcriptase (Invitrogen, USA) was used to perform reverse transcription using the DHR1 primer. The reaction was incubated at 50 °C for 30 min and 85 °C for 5 min. Next, 5 μL cDNA was used as the template for the first PCR and 2 μL first PCR product as the template for the second PCR with TransTaq® High Fidelity DNA polymerase (Beijing TransGen Biotech, Ltd., China) according to the manufacturer’s instructions. The first and second PCR reaction parameters both included an initial incubation at 95 °C for 9 min, followed by 39 cycles of 94 °C for 45 s, 42 °C for 45 s and 72 °C for 50 s, with a final incubation step at 72 °C for 10 min. Final PCR products were electrophoresed on a 1% agarose gel.

### Sequence analysis

All positive PCR products from clinical fecal samples and from experimental serum and fecal samples were purified using the QIAquick PCR Purification Kit (QIAGEN, USA) and sequenced using an ABI 3130 Genetic Analyzer automated sequencing system (Applied Biosystems, USA). Sequences were analyzed using BLAST searches (http://blast.ncbi.nlm.nih.gov/Blast.cgi).

### ELISA for avian HEV antibodies

Anti-avian HEV IgG antibodies were detected in both clinical and experimental serum samples using an indirect ELISA described previously by Zhao et al. [16]. Briefly, a purified truncated recombinant CaHEV capsid protein expressed in *Escherichia coli* was used as the coating antigen for the indirect ELISA. After the coated plates were blocked and washed, the serum samples (100 μL/well) were added into the wells and incubated for 1 h at room temperature (RT). After three washes, a horseradish peroxidase-goat anti-chicken IgG diluted 1:4000 (100 μL/well) was added to the wells and incubated for 1 h again at room temperature. After a final three washes, 3,3′,5,5′-tetramethylbenzidine (TMB) was added to each well and the plates were incubated in the dark for 15 min at RT. The colorimetric reaction was stopped by adding 3 M H2SO4 (50 μL/well) and the optical density (OD) values were read at 450 nm using an automated ELISA plate reader (Bio-Rad, USA). All sera were tested in at least duplicate wells.

### Statistical analyses

Data collection and analyses were performed using IBM SPSS Statistics 20 (IBM, USA). The student^’^s t-test was used to estimate the differences in avian HEV infection rate between caged and cage-free chickens. For determination of the liver weight to body weight ratio and histologic lesion scores in the experimental inoculated chickens, statistical analysis was performed as described previously [27]. *P* ≤ 0.05 was considered significant.

## Abbreviations

ELISA: Enzyme-linked immunosorbent assay
HEV: Hepatitis E virus
NC: Negative Control
RT-nPCR: Reverse Transcription-nested Polymerase Chain Reaction
Wpi: Week post inoculation
